# Vascular Normalization Was Associated with Colorectal Tumor Regression upon Anti-PD-L1 Combinational Therapy

**DOI:** 10.1155/2023/5867047

**Published:** 2023-03-17

**Authors:** Yan Zhang, Jiayan Gao, Yan He, Ziwei Qi, Long Qian, Wanpei Chen, Haiyan Xu, Yanhua Yue, Xunyuan Mao, Shuxin Guo, Yan Zhou, Shuru Zhou, Songbing Qin, Xueguang Zhang, Yuhui Huang

**Affiliations:** ^1^The Aoyang Cancer Institute, The Affiliated Aoyang Hospital of Jiangsu University, Zhangjiagang, 215617 Jiangsu, China; ^2^Hematology Center, Cyrus Tang Medical Institute, The Collaborative Innovation Center of Hematology, State Key Laboratory of Radiation Medicine and Protection, Soochow University, Suzhou, 215123 Jiangsu, China; ^3^Department of Anesthesiology, The Sixth Affiliated Hospital of Sun Yat-sen University, Guangzhou, 510655 Guangdong, China; ^4^Department of Medical Oncology, The Second People's Hospital of Lianyungang, Lianyungang, 222000 Jiangsu, China; ^5^Department of Hematology, The Third Affiliated Hospital of Soochow University, Changzhou, 213003 Jiangsu, China; ^6^Department of Radiotherapy, The First Affiliated Hospital of Soochow University, Suzhou, 215006 Jiangsu, China; ^7^Jiangsu Institute of Clinical Immunology & Jiangsu Key Laboratory of Clinical Immunology, The First Affiliated Hospital of Soochow University, Suzhou, 215006 Jiangsu, China

## Abstract

Anti-PD-L1 therapy exhibits durable efficacy, but only in a small fraction of cancer patients. The immunosuppressive tumor microenvironment (TME) is a crucial obstacle that impedes cancer immunotherapy. Here, we found that anti-PD-L1 therapy coupled with CD4^+^ T cell depletion induced colorectal tumor regression and vascular normalization, while monotherapy only retarded tumor growth without affecting the tumor vasculature. Moreover, simultaneous PD-L1 blockade and CD4^+^ T cell depletion eradicated intratumoral PD-L1^+^ lymphoid and myeloid cell populations, while additively elevating the proportions of CD44^+^CD69^+^CD8^+^, central memory CD44^+^CD62L^+^CD8^+^, and effector memory CD44^+^CD62L^−^CD8^+^ T cells, suggesting a reduction in immunosuppressive cell populations and the activation of CD8^+^ T cells in the TME. Moreover, anti-PD-L1 therapy reduced the proportions of intratumoral PD-L1^+^ immune cells and suppressed tumor growth in a CD8^+^ T cell dependent manner. Together, these results suggest that anti-PD-L1 therapy induces tumor vascular normalization and colorectal tumor regression via CD8^+^ T cells, which is antagonized by CD4^+^ T cells. Our findings unveil the positive correlation of tumor regression and vascular normalization in colorectal tumor models upon anti-PD-L1 therapy, providing a potential new strategy to improve its efficacy.

## 1. Introduction

Cancer immunotherapy has become the fourth standard cancer treatment modality. Immune checkpoint blockade therapy (ICB) is one of the major cancer immunotherapeutic approaches for solid cancer [1–3]. Immune checkpoints are a group of inhibitory molecules, including cytotoxic T-lymphocyte-associated protein 4 (CTLA4), programmed death 1 (PD1), and programmed death ligand 1 (PD-L1). Their physiological function is to suppress activated immune cells to prevent the damage of immune responses to normal tissues [4, 5]. Tumors “hijack” this protective immune mechanism to evade host immune surveillance and facilitate tumor growth and progression [2, 6]. Therefore, immune checkpoint therapy blocks inhibitory signaling to reactivate immune effectors to eradicate malignant cancer cells [7]. Anti-PD1 and anti-PD-L1 are two of the most commonly used immune checkpoint blockers in solid cancer [2, 3, 8].

ICB has been approved in multiple countries to treat approximately 18 different histologic types of cancer, including metastatic melanoma, lung cancer, Hodgkin's lymphoma, and metastatic colorectal cancer. In a phase III clinical trial of Durvalumab (anti-PD-L1) after chemoradiotherapy in stage III non-small-cell lung cancer (NSCLC), the median progression-free survival (PFS) was 16.8 (13.0-18.1) months in the Durvalumab group (10 mg/kg, every two weeks), compared with 5.6 (4.6-7.8) months in the placebo group, demonstrating the survival benefit of anti-PD-L1 therapy in NSCLC [9]. In a phase II clinical trial of Pembrolizumab (anti-PD1) in colorectal cancer patients with or without deficient mismatch repair (dMMR), the immune-related objective response rate (ORR) reached 40% (4 of 10 patients) in dMMR colorectal cancers while 0% (0 of 18 patients) in mismatch repair-proficient (pMMR) colorectal cancers [10]. In another phase II clinical trial with Dostarlimab (anti-PD1) monotherapy in stage II or III colorectal cancer patients with dMMR, a total of 12 patients who had completed treatment with at least 6 months of follow up showed a clinical complete response [11]. Together, these reports show that locally advanced colorectal cancer with dMMR and microsatellite instability-high (MSI-H) is highly sensitive to PD1/PD-L1 blockade therapy [12, 13]. However, only about 5% of colorectal cancer patients are dMMR and MSI-H. The majority of advanced colorectal cancers are pMMR and had an exceptionally low response rate to immune checkpoint therapy [14, 15]. Therefore, how to improve the efficacy of ICB in colorectal cancers with pMMR and low (MSI-L) is an urgent need.

Insight into the mechanisms of ICB may provide an opportunity to improve the efficacy of ICB. Anti-PD-L1 therapy was originally considered to block the interaction between PD-L1 and PD1 [16]. Further studies showed that PD-L1 can also bind with B7-1 (CD80), which is expressed by T cells, B cells, and dendritic cells (DCs) and is a ligand of CD28 and CTLA4 [16]. Moreover, PD-L1 is expressed in various types of cells, including tumor cells, myeloid cells, and T cells [16, 17]. What are the roles of PD-L1 in different types of cells? Current reports suggest that both tumor and host immune cell-derived PD-L1 contributes to immunosuppression [18–21].

To extend the long-term survival benefits to more cancer patients, researchers are also exploring better biomarkers and more effective combinational strategies [2, 3, 22, 23]. PD-L1 can be upregulated by interferon *γ* (IFN*γ*) in tumor cells [24]. During the early development of anti-PD1 therapy, the levels of PD-L1 in tumor cells were used as a biomarker to stratify cancer patients for PD1/PD-L1 blockade therapy [25, 26]. However, this biomarker is not very efficient. A recent study suggested that tumor vessel perfusion could be used to predict the responsiveness of anti-PD1 and anti-CTLA4 therapy [27]. The underlying mechanism is that the abnormal tumor vasculature creates a hypoxic tumor microenvironment (TME) [23, 28]. Hypoxia promotes the production and accumulation of immunosuppressive components within the TME [29–31]. Thus, the induction of tumor vessel normalization alleviates hypoxia in the TME and improves cancer immunotherapy [23, 28, 32, 33].

In this study, we found that in colorectal tumor models, simultaneous PD-L1 blockade and CD4^+^ T cell depletion induced tumor vascular normalization, which was positively correlated with tumor regression. Anti-PD-L1 therapy reduced the proportions of intratumoral PD-L1^+^ lymphoid and myeloid cell populations and induced tumor vascular normalization in a CD8^+^ T cell dependent manner, while CD4^+^ T cells impeded these effects. Our findings suggest a potential new strategy to improve anti-PD-L1 therapy in colorectal tumors by remodeling the tumor vasculature.

## 2. Methods

### 2.1. Mice

C57BL/6 and Balb/c mice were obtained from commercial vendors: the Vital River Laboratories (Beijing, China) and the Shanghai SLAC Laboratory Animal Center (Shanghai, China). All mice were maintained under specific pathogen-free condition with a 12 h light-dark cycle at a temperature of 21-23°C and humidity of 35-55% in the animal facility of Soochow University. For all experiments, female mice (6-8 weeks old) were used. All animal studies were approved by the Institutional Laboratory Animal Care and Use Committee of Soochow University. All of the procedures were performed in compliance with the Animal Care and Use Regulations of China.

### 2.2. Tumor Cell Lines

MCA38 and CT26 were considered as pMMR/MSI-L murien colorectal tumor models [34, 35]. The tumor cell lines were purchased from American Type Culture Collection. Cell lines were cultured in DMEM supplemented with 10% fetal bovine serum and 1% penicillin/streptomycin in a 5% CO_2_ incubator at 37°C. Cell lines were routinely tested for mycoplasma contamination, and only mycoplasma-negative cells were used for experiments.

### 2.3. Tumor Growth and Treatments

MCA38 and CT26 colon tumor cells (2 × 10^5^) were subcutaneously inoculated into the right flank of C57BL/6 and Balb/c mice, respectively. When tumors reached 4-5 mm in diameter (day 0), mice were randomized to different groups and treated intraperitoneally (*i.p.*) with IgG and anti-PD-L1 antibodies (10 mg/kg body weight, clone: 10F.9G2, catalog 124341, BioLegend) every 3 days from day 0 [36]. Tumor sizes were measured every three days from day 0 using a caliber. Tumor volumes were calculated using the formula (Lenth × Width^2^) × *π*/6. At the end of the experiments, the mice were euthanized and the tumor tissues were harvested for subsequent analyses.

### 2.4. Selective T Cell Depletion

MCA38 and CT26 colon tumors grew to around 4-5 mm in diameter (Day 0). Mice were randomly assigned to the indicated groups, and received anti-CD4 (clone: GK1.5, Bio X Cell) or anti-CD8 antibody (clone: 53-6.72, Bio X Cell) treatments (200 *μ*g/mouse, *i.p.* injection) on days 0, 2, and 8. At the end of the experiments, the efficiency of CD4^+^ and CD8^+^ T cell depletion was verified by flow cytometry analysis [27].

### 2.5. Flow Cytometry Analysis

Tumor tissues were harvested and digested in DMEM with collagenase type 1A (1.5 mg/ml), hyaluronidase (1.5 mg/ml) and DNase (20 U/ml) at 37°C for 45 minutes. After digestion, tissues were passed through 70 *μ*m cell strainers to obtain single cell suspensions. Single-cell suspensions were then incubated with a rat anti-mouse CD16/CD32 monoclonal antibody to block Fc*γ* receptors. After blocking, the cells were incubated with a flow cytometric antibody cocktail for 15 minutes in a refrigerator in the dark. The following antibodies were used for flow cytometry analysis: CD4-PE (Clone: GK1.5, catalog 100408), CD8a-FITC (Clone: 53-6.72, catalog 100706), CD4-Alexa Flour 700 (Clone: RM4-4, catalog 116022), PD-L1-PE (Clone: 10F.9G2, catalog 124308), CD44-APC (Clone: IM7, catalog 103012), CD69-PE-Cy7 (Clone: H1.2F3, catalog 104512), CD62L-APC-Cy7 (Clone: MEL-14, catalog 104428), F4/80-FITC (Clone: BM8, catalog123108), Gr1-APC-Cy7 (Clone: RB6-8C5, catalog108424), CD45-BV421 (Clone: 30-F11, catalog 103134), and CD11b-BV510 (Clone: M1/70, catalog 101263) (from BioLegend) [37, 38]. The reagent 7-aminoactinomycin D (7AAD, eBioscience) was added to stain dead cells (5 *μ*l/tube) just before flow cytometry analysis. Samples were analyzed on a Gallios flow cytometer (Beckman), and data were analyzed with Kaluza software (version 1.3).

### 2.6. Tumor Tissue Perfusion Analysis

Tumor tissue perfusion was evaluated by histologic analysis of the intravenous (*i.v.*) injection of Hoechst 33342 (Sigma-Aldrich), a cell permeable nucleic acid staining agent, as previously described [27, 38]. Briefly, five minutes after *i.v.* injection of Hoechst 33342 (10 mg/kg), mice were systemically perfused with PBS, and the tumors were removed and fixed with 4% paraformaldehyde. This procedure stained the perfused vessels and tumor area with fluorescent nucleus-bound Hoechst 33342. Images of tumors were collected with an Olympus FV3000 confocal laser-scanning microscope. Nonspecific nuclear staining (Sytox Green, S7020, Molecular Probes) was used to counterstain the slides. In each field, the mean fluorescence intensity of Hoechst 33342^+^ areas was calculated by using Image-Pro plus software (version 6.0).

### 2.7. Immunohistochemical Staining

Tumor tissues were fixed in 4% paraformaldehyde for 3 hours, followed by incubation in 30% sucrose overnight at 4°C. The tissues were OCT embedded and kept at -80°C. Staining for the endothelial cell marker CD31 (1 : 200, clone MEC13.3, catalog 550274, BD Biosciences) was performed on frozen sections (20 *μ*m thickness), followed by staining with secondary antibody Alexa Fluor 647 donkey anti-rat IgG (1 : 200, catalog 712-605-153, Jackson ImmunoResearch) in dark, humid chambers [27]. The slides were counterstained for cell nuclei with Sytox Green. Fluorescence images were obtained with an Olympus FV3000 confocal laser-scanning microscope. Microvessel density was assessed by using Image-Pro plus software (version 6.0). The variables were determined for 4-5 photographic areas from each tumor (640 × 640 *μ*m^2^ each). Confocal images were taken in randomly selected fields, excluding the tumor periphery.

### 2.8. Quantitative Real-Time PCR (RT-qPCR)

The purification and analysis of gene transcription by RT-qPCR were conducted as previously described [27, 38]. Briefly, total RNA was purified from CT26 tumor tissues using a MicroElute Total RNA kit (Omega). The cDNA was synthesized by a RevertAid First Strand cDNA Synthesis Kit (Thermo Scientific). The levels of mRNA transcription were measured by a high-throughput fluorescence quantitative PCR meter (LightCycler480 II) (Roche). B-Actin was used as a reference gene to calculate the differences in gene expression (fold change). The sequences of the primers were shown in Supplementary Table 1.

### 2.9. Statistical Analysis

Prism statistical software (version 8, GraphPad Software, Inc.) was used to perform statistical analyses. All of the data were presented as the mean ± standard error (SD). Data were first confirmed for their normal distribution using the Kolmogorov-Smirnov test. The differences between two groups were determined by using unpaired 2-tailed Student's *t*-tests. Comparisons among more than two groups were assessed by using one-way analysis of variance (ANOVA). A *P* value less than 0.05 was considered as statistically significant. *P* values lower than 0.05, 0.01, and 0.001 were indicated as “^∗^,” “^∗∗^,” and “^∗∗∗^,” respectively.

## 3. Results

### 3.1. The Antitumor Effects of PD-L1 Blockade Are Associated with a Reduction in Intratumoral PD-L1^+^ Immune Cells in the CT26 Colorectal Tumor Model

To understand the resistance mechanism of colorectal cancer patients to immune checkpoint therapies, we administrated anti-PD-L1 therapy to two commonly used pMMR/MSI-L murine colorectal tumor models CT26 and MCA38 [34, 35]. In the CT26 colorectal tumor model, anti-PD-L1 treatments retarded tumor growth (Supplementary Figure S1a). Interestingly, anti-PD-L1 treatments did not significantly change the proportions of intratumoral CD4^+^ and CD8^+^ T cells but significantly decreased the percentages of tumor-infiltrating PD-L1^+^ myeloid and lymphoid cells, compared to the control group (Supplementary Figure S1b, c, and S2). The data suggest that the antitumor effects of PD-L1 blockade therapy in CT26 colorectal tumor model are associated with a reduction in tumor-infiltrating PD-L1^+^ immune cells, but not the number of intratumoral T cells.

### 3.2. Simultaneous PD-L1 Blockade and CD4^+^ T Cell Depletion Induces CT26 Tumor Vascular Normalization, Which Is Associated with Tumor Regression

**Figure 1:**
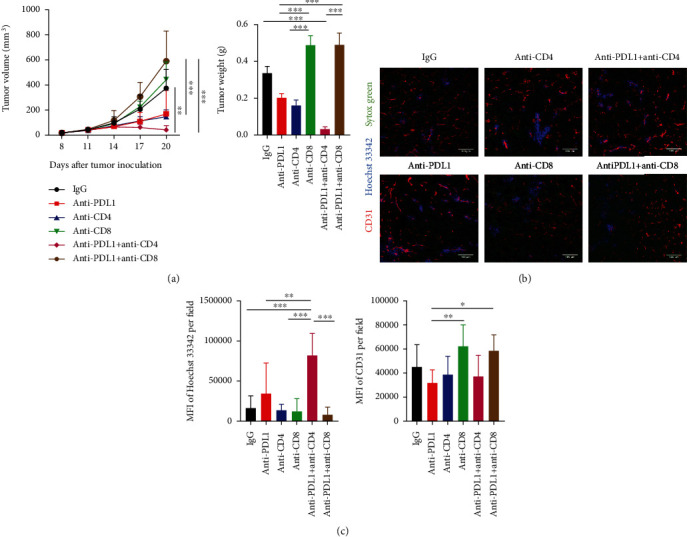
Simultaneous PD-L1 blockade and CD4^+^ T cell depletion induced vascular normalization and tumor regression in CT26 colorectal tumors. Balb/c mice were inoculated with 2 × 10^5^ CT26 colon tumor cells. When tumors were grown to 4-5 mm in diameter (day 0), mice were randomly assigned to 6 groups and treated with anti-CD8 and anti-CD4 antibodies on days -1, 1, and 8. On day 0, mice were treated with anti-PD-L1 (10 mg/kg) or IgG (10 mg/kg) antibody, every 3 days for 4 doses. On day 12 post anti-PD-L1 treatments, mice were injected with 200 *μ*g/mouse Hoechst 33342 via the tail vein. Five minutes later, tumors were harvested for immunohistochemistry staining. (a) Tumor growth curves and tumor weight. (b) Representative figures showed CD31^+^ tumor blood vessels (red) and Hoechst 33342 stained, functional blood vessels (blue) in CT26 tumor tissues. (c) Total tumor blood vessel density and Hoechst 33342 perfused area were quantified. Scale bars: 100 *μ*m. Significant differences were determined by one-way ANOVA. Data were from one experiment representative of two independent experiments with similar results (*n* = 7 − 8 mice per group). Data were shown as means ± SD. ^∗^*P* < 0.05, ^∗∗^*P* < 0.01, ^∗∗∗^*P* < 0.001.

Myeloid cells have been shown to suppress T cell activities via their PD-L1 expression [20, 36]. Indeed, our data showed that tumor growth inhibition by anti-PD-L1 therapy was correlated with reduced tumor-infiltrating PD-L1^+^ immune cells (Supplementary Figure S1). T cells, particularly CD8^+^ T cells, are considered as a major player in cancer immunotherapy; however, the roles of CD4^+^ and CD8^+^ T cells in anti-PD-L1 therapy are not very clear. To answer this question, we applied specific antibodies to selectively deplete CD4^+^ or CD8^+^ T cells upon PD-L1 blockade therapy. In the CT26 colorectal tumor model, anti-PD-L1 therapy or CD4^+^ T cell depletion alone retarded tumor growth, while CD8^+^ T cell depletion did not influence tumor growth (Figure 1(a) and Supplementary Figure S3). Interestingly, simultaneous PD-L1 blockade and CD4^+^ T cell depletion additively suppressed tumor growth, resulting in tumor regression, while CD8^+^ T cell depletion completely reversed the antitumor effects of anti-PD-L1 therapy (Figure 1(a) and Supplementary Figure S3). These results suggest that anti-PD-L1 therapy inhibits CT26 tumor growth in a CD8^+^ T cell dependent manner, while CD4^+^ T cells exhibit opposite effects. Since the hypoxic tumor microenvironment is a key obstacle impeding T cell stimulation [23, 39, 40], thus we postulated that simultaneous PD-L1 blockade and CD4^+^ T cell depletion promotes tumor vascular normalization. We adapted cell permeant nuclear acid staining agent Hoechst 33342 to evaluate tumor blood vessel perfusion *in vivo*. After tail vein injection of Hoechst 33342, we harvested tumor tissues and then performed immunohistochemistry staining. Neither anti-PD-L1 therapy nor depletion of CD4^+^ or CD8^+^ T cells alone changed tumor blood vessel perfusion compared to the control group, while simultaneous PD-L1 blockade and CD4^+^ T cell depletion significantly elevated tumor blood vessel perfusion compared to all of the other groups, indicating tumor vascular normalization (Figures 1(b) and 1(c)). Simultaneous PD-L1 blockade and CD8^+^ T cell depletion did not alter tumor blood vessel perfusion (Figures 1(b) and 1(c) and Supplementary Figure S3). Together, these results suggest that anti-PD-L1 therapy induces CT26 tumor vascular normalization and tumor regression via CD8^+^ T cells, while CD4^+^ T cells antagonize these effects.

### 3.3. Simultaneous PD-L1 Blockade and CD4^+^ T Cell Depletion Additively Reduces PD-L1^+^ Immune Cells and Activates CD8^+^ T Cells in CT26 Colorectal Tumors

**Figure 2:**
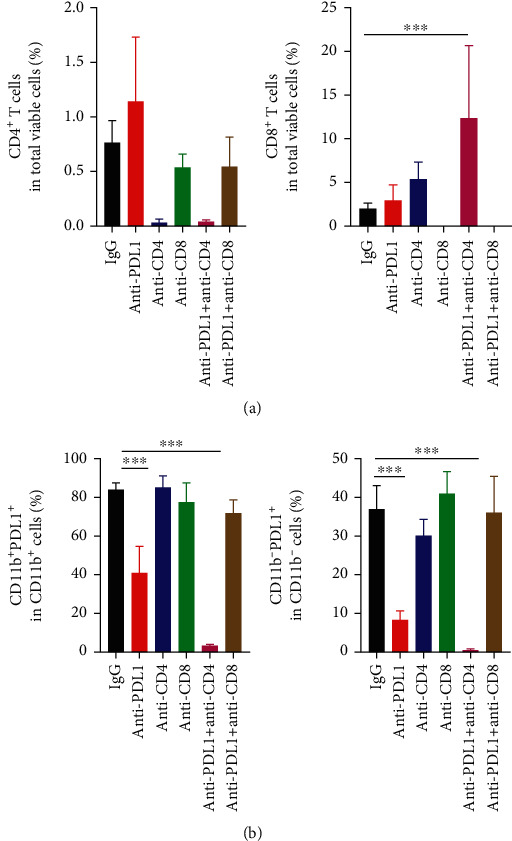
Anti-PD-L1 therapy decreased the proportions of intratumoral PD-L1^+^ immune cells in a CD8^+^ T cell dependent manner, while CD4^+^ T cells antagonized those effects. CT26 tumor-bearing mice were prepared and treated as described in Figure 1. On day 12 post anti-PD-L1 treatments, tumor tissues were harvested and analyzed by flow cytometry. (a) The proportions of intratumoral CD4^+^ and CD8^+^ T cells were analyzed by flow cytometry. (b) The percentages of intratumoral PD-L1^+^CD11b^−^ cells in lymphoid cells and PD-L1^+^CD11b^+^ cells in myeloid cells were assessed by flow cytometry. Significant differences were determined by one-way ANOVA. Data were from one experiment representative of two independent experiments with similar results (*n* = 7 − 8 mice per group). Data were shown as means ± SD. ^∗^*P* < 0.05, ^∗∗∗^*P* < 0.001.

Since the suppression of tumor growth by anti-PD-L1 therapy required CD8^+^ T cells and anti-PD-L1 treatments alone did not change the T cell number within the tumor parenchyma, we speculated that anti-PD-L1 therapy modulates T cell function. We then harvested CT26 tumor tissues and analyzed tumor-infiltrating immune cells. Anti-PD-L1 treatments did not change the percentages of intratumoral CD8^+^ T cells but significantly reduced the proportions of PD-L1^+^ myeloid and lymphoid cells, compared to the control groups (Figure 2(a) and Supplementary Figure S4, S5). *In vivo* depletion of CD4^+^ T cells significantly increased the percentages of intratumoral CD8^+^ T cells, but did not alter the proportions of PD-L1^+^ immune cells, compared to the control groups (Figure 2(b) and Supplementary Figure S6, S7). Notably, simultaneous PD-L1 blockade and CD4^+^ T cell depletion significantly increased the percentages of intratumoral CD8^+^ T cells and almost eradicated PD-L1^+^ myeloid and lymphoid cells, compared to the control and monotreatment groups (Figure 2(b) and Supplementary Figure S6, S7). *In vivo* depletion of CD8^+^ T cells completely reversed the effects of anti-PD-L1 therapy on PD-L1^+^ immune cells, compared to the control and anti-PD-L1 groups (Figure 2(b) and Supplementary Figure S6, S7). These results suggest that anti-PD-L1 therapy decreases PD-L1^+^ immune cells in a CD8^+^ T cell dependent manner, while CD4^+^ T cells impede these effects.

**Figure 3:**
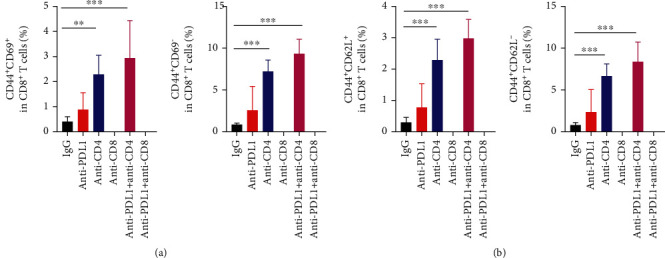
PD-L1 blockade and CD4^+^ T cell depletion additively promoted CD8^+^ T cell activation in tumor-draining lymph nodes. CT26 tumor-bearing mice were prepared and treated as described in Figure 1. On day 12 post anti-PD-L1 treatments, tumor-draining lymph nodes were harvested and analyzed by flow cytometry. (a) The percentages of CD44^+^CD69^+^ and CD44^+^CD69^−^ cells in the CD8^+^ T cell population. (b) The proportions of CD44^+^CD62L^+^ and CD44^+^CD62L^−^ cells in CD8^+^ T cell population. Significant differences were determined by one-way ANOVA. Data were from one experiment representative of two independent experiments with similar results (*n* = 7 − 8 mice per group). Data were shown as means ± SD. ^∗∗^*P* < 0.01, ^∗∗∗^*P* < 0.001.

**Figure 4:**
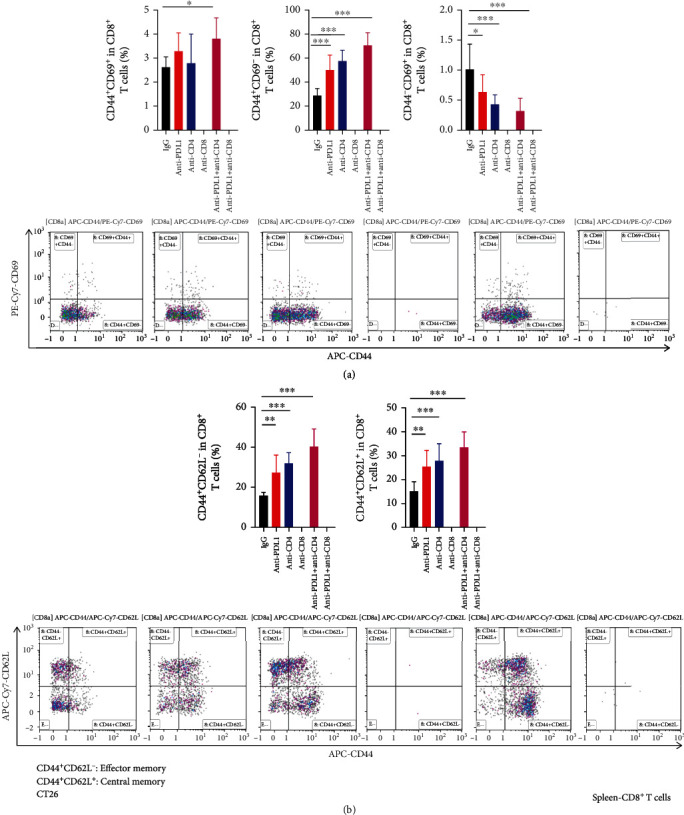
PD-L1 blockade and CD4^+^ T cell depletion additively promoted splenic CD8^+^ T cell activation. CT26 tumor-bearing mice were prepared and treated as described in Figure 1. On day 12 post anti-PD-L1 treatments, spleens were harvested for flow cytometric analysis. (a) The percentages of CD44^+^CD69^+^, CD44^+^CD69^−^, and CD44^−^ CD69^+^ cells in the CD8^+^ T cell population as well as representative flow figures were shown. (b) The percentages of CD44^+^CD62L^−^ and CD44^+^CD62L^+^ cells in the CD8^+^ T cell population as well as representative flow figures were shown. Significant differences were determined by one-way ANOVA (*n* = 7 − 8 mice per group). Data were shown as means ± SD. ^∗^*P* < 0.05, ^∗∗^*P* < 0.01, ^∗∗∗^*P* < 0.001.

We also analyzed the function of T cells in the tumor parenchyma and peripheral immune organs. The combination of CD4^+^ T cell depletion and PD-L1 blockade significantly increased the proportion of CD44^+^CD69^+^CD8^+^ T cells in CT26 tumor tissues (Supplementary Figure S8). We also analyzed the transcription of genes related to antitumor immunity in CT26 tumor tissues. PD-L1 blockade combined with CD4^+^ T cell depletion significantly elevated the transcription levels of the cytotoxic genes *Ifng* and *Tnfa*, while the immunosuppressive genes *Il10* and *Tgfb* were not different compared with the control or anti-PD-L1 alone group (Supplementary Figure S9). These results suggest that simultaneous PD-L1 blockade and CD4^+^ T cell depletion and PD-L1 blockade induce intratumoral CD8^+^ T cell activation. In the tumor-draining lymph nodes, CD4^+^ T cell depletion or its combination with PD-L1 blockade therapy significantly elevated the proportions of CD44^+^CD69^+^CD8^+^, as well as central memory CD44^+^CD62L^+^CD8^+^ and effector memory CD44^+^CD62L^−^CD8^+^ T cells (Figure 3 and Supplementary Figure S10, S11). In the spleen, PD-L1 blockade, CD4^+^ T cell depletion or their combination increased the proportions of CD44^+^CD69^−^CD8^+^ T cells compared with the control group (Figure 4(a)). The combination of CD4^+^ T cell depletion and PD-L1 blockade increased the proportions of CD44^+^CD69^+^CD8^+^ T cells compared with control group (Figure 4(a)). Moreover, PD-L1 blockade, CD4^+^ T cell depletion or their combination increased the proportions of central memory CD44^+^CD62L^+^CD8^+^and effector memory CD44^+^CD62L^−^CD8^+^ T cells, compared with the control group (Figure 4(b)). Together, these data suggest that PD-L1 blockade combined with CD4^+^ T cell depletion additively activates CD8^+^ T cells. Taken together, these results suggest that the combination of anti-PD-L1 treatments and CD4^+^ T cell depletion may inhibit CT26 tumor growth via the activation of CD8^+^ T cells and the reduction of immunosuppressive PD-L1^+^ immune cells.

### 3.4. Simultaneous PD-L1 Blockade and CD4^+^ T Cell Depletion Induces MCA38 Tumor Vascular Normalization, Which Is Associated with Tumor Regression

**Figure 5:**
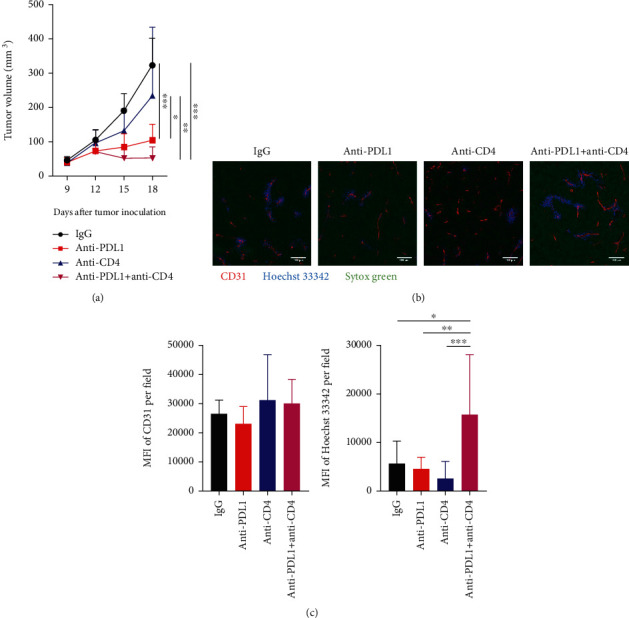
PD-L1 blockade with concurrent CD4^+^ T cell depletion induced vascular normalization and tumor regression in MCA38 colorectal tumors. C57BL/6 J mice were inoculated with 2 × 10^5^ MCA38 colon tumor cells. When MCA38 colon tumors reached 4-5 mm in diameter (day 0), mice were randomly assigned to 4 groups and treated with anti-CD4 antibody on days -1, 1, and 8. On day 0, mice were treated with anti-PD-L1 (10 mg/kg) or IgG (10 mg/kg) antibody, every 3 days for 3 doses. On day 9 post anti-PD-L1 treatments, mice were intravenously injected with 200 *μ*g/mouse Hoechst 33342, and tumor tissues were harvested after 5 minutes of the injection. (a) Tumor volumes were shown in each time point for each group. (b) Representative figures showing Hoechst 33342-stained areas (blue) and immunohistochemical staining of CD31 (red). Scale bars: 100 *μ*m. (c) Total tumor blood vessel density and Hoechst 33342 perfused area were quantified. Significant differences were determined by one-way ANOVA. Data were from one experiment representative of two independent experiments with similar results (*n* = 6 − 8 mice per group). Data were shown as means ± SD. ^∗^*P* < 0.05, ^∗∗^*P* < 0.01, ^∗∗∗^*P* < 0.001.

Thus far, our data have shown that CD4^+^ T cells hinder the effects of anti-PD-L1 therapy on either tumor growth or the tumor immune microenvironment in the CT26 colorectal tumor model. To determine whether this is unique in the CT26 tumor model, we performed a similar treatment regimen in another murine colorectal tumor model MCA38. Anti-PD-L1 therapy retarded tumor growth, while CD4^+^ T cell depletion alone did not significantly affect tumor growth (Figure 5(a) and Supplementary Figure S12). Notably, the combination of PD-L1 blockade and CD4^+^ T cell depletion induced tumor regression compared to the control group (Figure 5(a)). We then analyzed the tumor vasculature. Although PD-L1 blockade or CD4^+^ T cell depletion alone had no impact on tumor vessel perfusion compared with control group, concurrent PD-L1 blockade and CD4^+^ T cell depletion significantly improved tumor blood vessel perfusion compared with all the other groups in the MCA38 colorectal tumor model (Figures 5(b) and 5(c) and Supplementary Figure S12). These results suggest that intratumoral CD4^+^ T cells compromise the effects of anti-PD-L1 therapy on tumor regression and vascular normalization in the MCA38 colorectal tumor model.

### 3.5. Simultaneous PD-L1 Blockade and CD4^+^ T Cell Depletion Additively Increases Intratumoral CD8^+^ T Cells and Reduces PD-L1^+^ Immune Cells in MCA38 Colorectal Tumors

**Figure 6:**
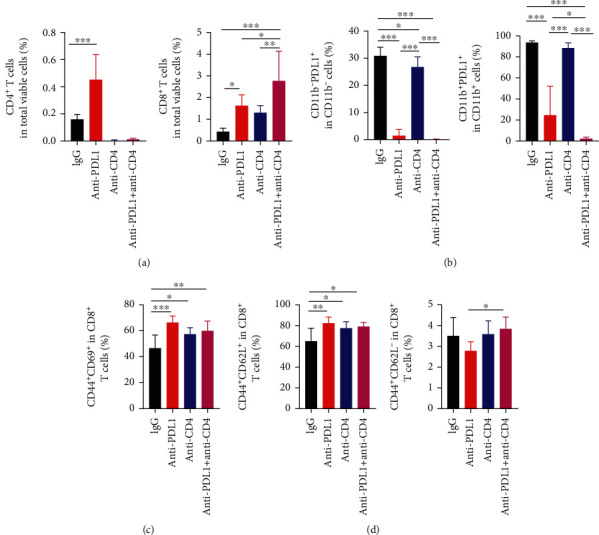
PD-L1 blockade and CD4^+^ T cell depletion additively promoted CD8^+^ T cell tumor infiltration and decreased the proportions of PD-L1^+^ immune cell populations within MCA38 tumors. MCA38 tumor-bearing mice were prepared and treated as described in Figure 5. On day 9 post anti-PD-L1 treatments, Mice were sacrificed and tumor tissues were harvested for flow cytometric analysis. (a) The percentages of intratumoral CD4^+^ and CD8^+^ T cells were assessed by flow cytometry. (b) The percentages of intratumoral PD-L1^+^CD11b^−^ and PD-L1^+^CD11b^+^ cells in lymphoid and myeloid cell populations, respectively. (c) The percentages of CD44^+^CD69^+^ cells in the CD8^+^ T cell population. (d) The percentages of CD44^+^CD62L^+^ and CD44^+^CD62L^−^ cells in the CD8^+^ T cell population. Significant differences were determined by one-way ANOVA. Data were from one experiment representative of two independent experiments with similar results (*n* = 6 − 8 mice per group). Data were shown as means ± SD. ^∗^*P* < 0.05, ^∗∗^*P* < 0.01, ^∗∗∗^*P* < 0.001.

Next, we analyzed the impacts of simultaneous PD-L1 blockade and CD4^+^ T cell depletion on the tumor immune microenvironment. Consistent with the tumor growth inhibition, anti-PD-L1 therapy promoted the tumor accumulation of CD8^+^ T cells and the combination of PD-L1 blockade and CD4^+^ T cell depletion further increased the proportion of intratumoral CD8^+^ T cells (Figure 6(a) and Supplementary Figure S13, S14). Consistent with the CT26 tumor model, anti-PD-L1 treatments significantly reduced the proportions of PD-L1^+^ lymphoid and myeloid cells compared with the control group (Figure 6(b)). Although CD4^+^ T cell depletion alone did not change the percentages of PD-L1^+^ immune cells, the combination of PD-L1 blockade and CD4^+^ T cell depletion eradicated PD-L1^+^ lymphoid and myeloid cells (Figure 6(b)). Moreover, PD-L1 blockade, CD4^+^ T cell depletion, or their combination not only increased the proportions of tumor-infiltrating CD44^+^CD69^+^CD8^+^ T cells but also elevated the proportions of central memory CD44^+^CD62L^+^CD8^+^ and effector memory CD44^+^CD62L^−^CD8^+^ T cells, compared with the control group (Figures 6(c) and 6(d)). Together, these data suggest that the addition of CD4^+^ T cell depletion to PD-L1 blockade therapy promotes the tumor infiltration of CD8^+^ T cells and the eradication of PD-L1^+^ immune cells in MCA38 tumors.

## 4. Discussion

Although PD-L1 blockade therapy has been approved for use in multiple types of cancer, the response rates are low, especially in colorectal cancer [10–15]. The underlying resistance mechanisms are complicated and require further investigation. Previous reports suggest a critical role of tumor-infiltrating myeloid cells in PD1/PD-L1 blockade therapy [20, 21, 36]. Our study showed that anti-PD-L1 therapy decreased the proportions of PD-L1^+^ lymphoid and myeloid cells, which corresponded to slow tumor growth. Simultaneous PD-L1 blockade and CD4^+^ T cell depletion activated CD8^+^ T cells and induced colorectal tumor regression, which was positively correlated with the induction of tumor vascular normalization. The depletion of CD8^+^ T cells reversed the reduction of PD-L1^+^ immune cells, tumor vascular normalization, and tumor growth inhibition induced by anti-PD-L1 therapy. These results suggest that PD-L1 blockade inhibits tumor growth in a CD8^+^ T cell dependent manner, while CD4^+^ T cells exert opposite effects. Thus, this study emphasizes CD8^+^ T cells as a major immune effector while CD4^+^ T cells as a major immune suppressor in anti-PD-L1 therapy in colorectal tumors.

The upregulation of PD-L1 in the tumor parenchyma represents a major mechanism of tumor immune evasion. Inflammatory cytokines, especially IFN*γ*, or oncogenic signaling pathways, such as phosphatase tensin homologs (PTEN) and epidermal growth factor receptor (EGFR), can induce PD-L1 expression in immune cells and malignant cells [16, 22, 24]. Therefore, the levels of PD-L1 expression serve as an important biomarker to stratify cancer patients and to predict the response to PD1/PD-L1 blockade therapy [25, 26]. Several studies have demonstrated that myeloid cells, especially DCs, hinder antitumor immune responses via high expression of PD-L1 [20, 21, 36]. PD-L1 can directly bind to PD1 in T cells and cause their dysfunction. PD-L1 may also bind with B7-1 and prevent the interaction between B7-1 and CD28, thus impeding T cell priming and stimulation. Our study found that anti-PD-L1 antibody treatments significantly decreased the proportions of PD-L1^+^ immune cell populations. The depletion of CD4^+^ T cells further prompted the reduction in PD-L1^+^ immune cell populations, especially PD-L1^+^ myeloid cells, upon anti-PD-L1 therapy. This may explain why CD4^+^ T cell depletion improved T cell activation and promoted tumor regression upon anti-PD-L1 therapy. However, we did not know how anti-PD-L1 antibody treatments or CD4^+^ T cell depletion caused the reduction in PD-L1^+^ immune cell populations. The isotype of the anti-PD-L1 antibody used in this study is IgG2b, which possesses a low ability to induce antibody-dependent cellular cytotoxicity (ADCC) and complement-dependent cytotoxicity (CDC). Consistently, the overall percentages of the major lymphoid and myeloid cell populations did not decrease in the anti-PD-L1 or anti-PD-L1 plus CD4^+^ T cell depletion groups compared to the control group. Even though we could not rule out the possibility that anti-PD-L1 antibody treatments promote immune cell tumor infiltration and simultaneously exert antibody-dependent cell-mediated cytotoxicity on PD-L1^+^ immune cells, the exact underlying mechanism requires further investigation.

T cells are major immune effectors and play crucial roles in cancer immunotherapy [2, 27, 38, 41], but their exact functions in antitumor immunity are not fully clarified and are sometimes even controversial. In the tumor microenvironment, T cells are highly heterogeneous, consisting of many subtypes of CD4^+^ and CD8^+^ T cells. CD8^+^ T cells are usually considered as cytotoxic T cells (CTLs), a major killer of malignant cells. CD4^+^ T cells can facilitate CD8^+^ T cell function when it is at the status of helper-1 T cells (Th1) or suppress CD8^+^ T cell activity when it is differentiated to regulatory T cells (Tregs). Several studies have shown that CD8^+^ T cells mediate the antitumor effects of anti-CTLA4 and anti-PD1 therapy [27, 41]. Here, we further showed that anti-PD-L1 therapy inhibited tumor growth in a CD8^+^ T cell dependent manner, and CD4^+^ T cells often exerted opposite effects. However, a recent study suggests that CTLA4 and PD1 blockade therapy inhibit tumor growth via CD4^+^ T cells [42]. This discrepancy could be due to the use of Matrigel to inoculate tumor cells and the CD4^−/−^ mouse model, which could alter the tumor microenvironment and disrupt the interactions occurring between CD4^+^ and CD8^+^ T cells during tumor initiation and progression. One remaining question is whether the opposite effects of CD4^+^ T cells on anti-PD-L1 therapy are mediated by PD-L1 expressed in CD4^+^ T cells or by its subtype Tregs. The exact mechanism requires further studies by establishing mouse model with PD-L1 specific knockout in CD4^+^ T cells and by using Foxp3-DTR mice to selectively deplete Tregs.

Successful cancer immunotherapy requires not only the stimulation of tumor antigen-specific immune responses but also an immunosupportive tumor microenvironment to sustain antitumor immunity [23, 43–45]. One of the critical characteristics of the tumor microenvironment is hypoxia as well as the resulting immunosuppression [29, 46]. Alleviating tumor tissue hypoxia via the induction of tumor vascular normalization improves the efficacy of cancer immunotherapy [32, 33, 38, 47, 48]. In both CT26 and MCA38 murine colorectal tumor models, we found that only the combination of PD-L1 blockade and CD4^+^ T cell depletion could induce tumor vascular normalization and tumor regression, indicating the contribution of vascular normalization to elicit durable antitumor immunity. This is consistent with our previous finding that the extent of ICB-induced tumor vascular normalization is positively correlated with its responsiveness [23, 27].

In conclusion, anti-PD-L1 therapy induces tumor vascular normalization, decreases the proportions of intratumoral PD-L1^+^ lymphoid and myeloid cells, and inhibits colorectal tumor growth in a CD8^+^ T cell dependent manner, while CD4^+^ T cells antagonize those effects. Moreover, tumor regression by the combination of PD-L1 blockade and CD4^+^ T cell depletion is positively correlated with tumor vascular normalization in colorectal tumor models. Thus, our findings reveal the opposite roles of CD4^+^ and CD8^+^ T cells in anti-PD-L1 therapy and suggest a potential new strategy to potentiate anti-PD-L1 therapy by remodeling tumor blood vessels in colorectal tumors.

## Data Availability

All data used to support the results are included in the article and supplementary files.

## Supplementary Materials

Supplementary Materials Supplementary Figure S1: anti-PD-L1 therapy retarded CT26 tumor growth and decreased the proportions of PD-L1^+^ immune cell populations. Figure S2: the gating strategies for Figure S1. Figure S3: simultaneous PD-L1 blockade and CD4^+^ T cell depletion induced vascular normalization and tumor regression in CT26 colorectal tumors. Figure S4: the gating strategies for Figures 2, S6, S8. Figure S5: representative flow cytometry plots of Figure 2. Figure S6: PD-L1 blockade and CD4^+^ T cell depletion additively decreased the proportions of PD-L1^+^ lymphoid and myeloid cell populations within CT26 tumor tissues. Figure S7: representative flow cytometry plots of Figure S6. Figure S8: the combination of PD-L1 blockade and CD4^+^ T cell depletion activated tumor-infiltrating CD8^+^ T cells in CT26 tumors. Figure S9: the combination of PD-L1 blockade and CD4^+^ T cell depletion elevated the transcription of proinflammatory genes. Figure S10: the gating strategies for Figure 3. Figure S11: representative flow cytometry plots of Figure 3. Figure S12: PD-L1 blockade with concurrent CD4^+^ T cell depletion induced vascular normalization and tumor regression in MCA38 colorectal tumors. Figure S13: the gating strategies for Figure 6. Figure S14: the representative flow cytometry plots of Figure 6. Table 1: the sequences of primers for RT-qPCR.
