# Supplementary material for: Vascular Normalization Was Associated with Colorectal Tumor Regression upon Anti-PD-L1 Combinational Therapy
Source: J Immunol Res. 2023 Mar 17;2023:5867047. doi: 10.1155/2023/5867047 (PMC10038742; doi:10.1155/2023/5867047)
Supplement: Supplementary Materials — Supplementary Figure S1: anti-PD-L1 therapy retarded CT26 tumor growth and decreased the proportions of PD-L1+ immune cell populations. Figure S2: the gating strategies for Figure S1. Figure S3: simultaneous PD-L1 blockade and CD4+ T cell depletion induced vascular normalization and tumor regression in CT26 colorectal tumors. Figure S4: the gating strategies for Figures 2, S6, S8. Figure S5: representative flow cytometry plots of Figure 2. Figure S6: PD-L1 blockade and CD4+ T cell depletion additively decreased the proportions of PD-L1+ lymphoid and myeloid cell populations within CT26 tumor tissues. Figure S7: representative flow cytometry plots of Figure S6. Figure S8: the combination of PD-L1 blockade and CD4+ T cell depletion activated tumor-infiltrating CD8+ T cells in CT26 tumors. Figure S9: the combination of PD-L1 blockade and CD4+ T cell depletion elevated the transcription of proinflammatory genes. Figure S10: the gating strategies for Figure 3. Figure S11: representative flow cytometry plots of Figure 3. Figure S12: PD-L1 blockade with concurrent CD4+ T cell depletion induced vascular normalization and tumor regression in MCA38 colorectal tumors. Figure S13: the gating strategies for Figure 6. Figure S14: the representative flow cytometry plots of Figure 6. Table 1: the sequences of primers for RT-qPCR. [file 5867047.f1.docx]

**Vascular normalization was associated with colorectal tumor regression upon anti-PD-L1 combinational therapy**

Yan Zhang^1,2^, Jiayan Gao^1^, Yan He^2^, Ziwei Qi^2^, Long Qian^2^, Wanpei Chen^2,3^, Haiyan Xu^2,4^, Yanhua Yue^2,5^, Xunyuan Mao^2^, Shuxin Guo^2^, Yan Zhou^1^, Shuru Zhou^1^, Songbing Qin^6,*^, Xueguang Zhang^7^, Yuhui Huang^2,*^

| **A table of contents** | |
| --- | --- |
| **1** | **Figure S1.** Anti-PD-L1 therapy retarded CT26 tumor growth and decreased the proportions of PD-L1^+^ immune cell populations. |
| **2** | **Figure S2.** The gating strategies for Fig S1. |
| **3** | **Figure S3.** Simultaneous PD-L1 blockade and CD4^+^ T cell depletion induced vascular normalization and tumor regression in CT26 colorectal tumors. |
| **4** | **Figure S4.** The gating strategies for Figs 2, S6, S8. |
| **5** | **Figure S5.** The representative flow cytometry plots of Fig 2. |
| **6** | **Figure S6.** PD-L1 blockade and CD4^+^ T cell depletion additively decreased the proportions of PD-L1^+^ lymphoid and myeloid cell populations within CT26 tumor tissues. |
| **7** | **Figure S7.** The representative flow cytometry plots of Fig S6. |
| **8** | **Figure S8.** The combination of PD-L1 blockade and CD4^+^ T cell depletion activated tumor-infiltrating CD8^+^ T cells in CT26 tumors. |
| **9** | **Figure S9.** The combination of PD-L1 blockade and CD4^+^ T cell depletion elevated the transcript of pro-inflammatory genes. |
| **10** | **Figure S10.** The gating strategies for Fig 3. |
| **11** | **Figure S11.** The representative flow cytometry plots of Fig 3. |
| **12** | **Figure S12.** PD-L1 blockade with concurrent CD4^+^ T cell depletion induced vascular normalization and tumor regression in MCA38 colorectal tumors. |
| **13** | **Figure S13.** The gating strategies for Fig 6. |
| **14** | **Figure S14.** The representative flow cytometry plots of Fig 6. |

**Supplementary figures**

| **** |
| --- |

**Fig. S1 Anti-PD-L1 therapy retarded CT26 tumor growth and decreased the proportions of PD-L1^+^ immune cell populations.**

Balb/c mice were inoculated with 2🞨10^5^ CT26 colon tumor cells. When tumors were grown to 4-5 mm in diameter, mice were randomly assigned to 2 groups and treated with an anti-PD-L1 antibody (10 mg/kg) or IgG antibody (10 mg/kg) every 3 days for 3 doses. **(A)** Tumor growth curves. **(B)** The percentages of tumor-infiltrating CD4^+^ and CD8^+^ T cells were analyzed by flow cytometry. **(C)** The percentages of tumor-infiltrating PD-L1^+^CD11b^-^ cells in lymphoid cells and PD-L1^+^CD11b^+^ cells in myeloid cells were assessed by flow cytometry. Significance was determined by Student’s *t*-test, two-tailed (*n* = 6-7 mice per group). Data were shown as means ± SD. **P* < 0.05, ****P* < 0.001.

| **** |
| --- |
| **Fig. S2 The gating strategies for Fig S1.** |

| **** |
| --- |

**Fig. S****3 Simultaneous PD-L1 blockade and CD4^+^ T cell depletion induced vascular normalization and tumor regression in CT26 colorectal tumors.**

Balb/c mice were inoculated with 2🞨10^5^ CT26 colon tumor cells. When tumors were grown to 4-5 mm in diameter (Day 0), mice were randomly assigned to 6 groups and treated with anti-CD8 and anti-CD4 antibodies on days -1, 1, and 8. On day 0, mice were treated with anti-PD-L1 (10 mg/kg) or IgG (10 mg/kg) antibody, every 3 days for 4 doses. On day 12 post anti-PD-L1 treatments, mice were injected with 200 μg/mouse Hoechst 33342 via tail vein. Five minutes later, tumors were harvested for immunohistochemistry staining. **(A)** Tumor growth curves and tumor weight. **(B)** Total tumor blood vessel density and Hoechst 33342 perfused area were quantified. Representative figures showed CD31^+^ tumor blood vessels (red) and Hoechst 33342 stained, functional blood vessels (blue) in CT26 tumor tissues. Scale bars: 100 μM. Significant difference was determined by one-way ANOVA (*n* = 7-8 mice per group). Data were shown as means ± SD. **P* < 0.05, ***P* < 0.01, ****P* < 0.001.

| **** |
| --- |
| **Fig. S4 The gating strategies for Figs 2, S6, S8.** |

| **** |
| --- |
| **Fig. S5 The representative flow cytometry plots of Fig 2.** |

| **** |
| --- |

**Fig. S6 PD-L1 blockade and CD4^+^ T cell depletion additively decreased the proportions of PD-L1^+^ lymphoid and myeloid cell populations within CT26 tumor tissues.**

Balb/c mice were inoculated with 2🞨10^5^ CT26 colon tumor cells. When tumor sizes reached 4-5 mm in diameter (Day 0), mice were randomly divided into 6 groups and treated with anti-CD8 and anti-CD4 antibodies on days 0, 2, and 8. On day 1, mice were started to treat with an anti-PD-L1 antibody (10 mg/kg) or IgG antibody (10 mg/kg) every 3 days for 4 doses. On day 12 post anti-PD-L1 treatments, tumors were harvested for flow analysis. **(A)** The percentages of CD4^+^PD-L1^+^ cells in CD4^+^ T cell population. **(B)** The percentages of CD8^+^PD-L1^+^ cells in CD8^+^ T cell population. **(C)** The percentages of F4/80^+^PD-L1^+^/F4/80^+^ and Gr1^int^F4/80^int^PD-L1^+^/Gr1^int^F4/80^int^. Significant difference was determined by one-way ANOVA (*n* = 7-8 mice per group). Data were shown as means ± SD. **P* < 0.05, ****P* < 0.001.

| **** |
| --- |
| **Fig. S7 The representative flow cytometry plots of Fig S6.** |

| **** |
| --- |

**Fig. S8 The combination of PD-L1 blockade and CD4^+^ T cell depletion activated tumor-infiltrating CD8^+^ T cells in CT26 tumors.**

CT26 tumor-bearing mice were prepared and treated as described in Fig S6. On day 12 post anti-PD-L1 treatments, tumors were harvested and CD44^+^CD69^+^ and CD44^+^CD69^-^ cells in tumor-infiltrating CD8^+^ T cell population were assessed by flow cytometry. Significant difference was determined by one-way ANOVA (*n* = 7-8 mice per group). Data were shown as means ± SD. ***P* < 0.01.

| **** |
| --- |

**Fig. S9 The combination of PD-L1 blockade and CD4^+^ T cell depletion elevated the transcript of pro-inflammatory genes.**

CT26 tumor-bearing mice were prepared and treated as described in Fig S6. On day 12 post anti-PD-L1 treatments, tumor tissues swere harvested for RT-qPCR analysis. Significant difference was determined by one-way ANOVA (*n* = 4-6 mice per group). Data were shown as means ± SD. **P* < 0.05, ***P* < 0.01. ****P* < 0.001.

| **** |
| --- |
| **Fig. S10 The gating strategies for Fig 3.** |

| **** |
| --- |
| **Fig. S11 The representative flow cytometry plots of Fig 3.** |

| **** |
| --- |
| **Fig. S12 PD-L1 blockade with concurrent CD4^+^ T cell depletion induced vascular normalization and tumor regression in MCA38 colorectal tumors.**  C57BL/6J mice were inoculated with 2🞨10^5^ MCA38 colon tumor cells, When MCA38 colon tumors reached 4-5 mm in diameter (Day 0), mice were randomly assigned to 4 groups and treated with anti-CD4 antibody on days -1, 1, and 8. On day 0, mice were started to treat with anti-PD-L1 (10mg/kg) or IgG (10 mg/kg) antibody, every 3 days for 3 doses. On day 9 post anti-PD-L1 treatments, mice were intravenously injected with 200 μg/mouse Hoechst 33342 and tumor tissues were harvested after 5 minutes of the injection. **(A)** Tumor volumes and tumor weight. **(B)** Total tumor blood vessel density and Hoechst 33342 perfused area were quantified. Significant difference was determined by one-way ANOVA (*n* = 7-8 mice per group). Data were shown as means ± SD. **P* < 0.05, ***P* < 0.01, ****P* < 0.001. |

| **** |
| --- |
| **Fig. S13 The gating strategies for Fig 6.** |

| **** |
| --- |
| **Fig. S14 The representative flow cytometry plots of Fig 6.** |

**Supplementary Table 1.** The sequences of primers for RT-qPCR.

| Gene | Primer | Sequence (5’-3’) |
| --- | --- | --- |
| *B-actin* | Forward Reverse | ATCGTGCGTGACATCAAAGA ACAGGATTCCATACCCAAGAAG |
| *Il10* | Forward Reverse | CCAGAGCCACATGCTCCTA AGGGGAGAAATCGATGACAG |
| *Ifng* | Forward Reverse | CCAAGTTTGAGGTCAACAACCC GGGACAATCTCTTCCCCACC |
| *Tnfa* | Forward Reverse | CCGATGGGTTGTACCTTGTC  CGGACTCCGCAAAGTCTAAG |
| *Nos2* | Forward Reverse | CTGCACCGAAGATATCTTCA  CTGCACCGAAGATATCTTCA |
| *Vegfa* | Forward Reverse | CGGGCCTCGGTTCCAG  TGAACTTGATCACTTCATGGGACT |
| *Ang1* | Forward Reverse | ATGGAAAATTATACTCAGTGGCTGC  ATTTAGTACCTGGGTCTCAACATC |
| *Ang2* | Forward Reverse | TACAAAGAGGGCTTCGGGAG  GTTGGACTCTTCACCAGCGA |
| *Timp1* | Forward Reverse | GAGACACACCAGAGCAGATACC  GCTGGTATAAGGTGGTCTCGT |
| *Tgfb* | Forward Reverse | GCTGAACCAAGGAGACGGAAT  GCCTTAGTTTGGACAGGATCTG |
| *Pf4* | Forward Reverse | AGCTCATAGCCACCCTGAAGA  ACAATTGACATTTAGGCAGCTGAT |
